# Development, Analysis, and Determination of Pharmacokinetic Properties of a Solid SMEDDS of Voriconazole for Enhanced Antifungal Therapy

**DOI:** 10.3390/life14111417

**Published:** 2024-11-02

**Authors:** Hitesh Kumar Dewangan, Rajiv Sharma, Kamal Shah, Perwez Alam

**Affiliations:** 1 University Institute of Pharma Sciences (UIPS), Chandigarh University, NH-95 Chandigarh Ludhiana Highway, Mohali 140413, Punjab, India; 2 Institute of Pharmaceutical Research (IPR), GLA University Mathura, NH-2 Delhi Mathura Road, Po-Chaumuhan, Chaumuhan 281406, Uttar Pradesh, India; 3 Department of Pharmacognosy, College of Pharmacy, King Saud University, P.O. Box 2457, Riyadh 11451, Saudi Arabia; aperwez@ksu.edu.sa

**Keywords:** voriconazole, SEDDS, emulsion, microemulsion, formulation

## Abstract

Background: Voriconazole is an antifungal drug, which is classified under Bio-Classification System-II and has low water solubility (0.71 mg/mL) and high permeability. Hardly any endeavors have been made to increase the bioavailability of voriconazole. Objective: To develop and evaluate a solid SMEDDS (self-microemulsifying drug delivery system) for antifungal activity. Methods: Based on solubility studies of Labrafil-M 1994 CS (oil), Cremophor-RH 40 (a surfactant) and Transcutol-HP (a co-surfactant) were selected as components of the SMEDDS and a pseudo-ternary phase diagram was prepared. Thereafter, the oil, surfactant, and co-surfactant were mixed with altered weight ratios (1:1/1:2/2:1) and evaluated through various in vitro, in vivo analyses. Results: The particle size of the optimized formulation was observed to be 19.04 nm and the polydispersity index (PDI) value was found to be 0.162 with steady-state zeta potential. The optimized liquid SMEDDS was converted into a solid SMEDDS. Various adsorbents, such as Aerosil-200, Avicel-PH101, Neusilin-US2, and Neusilin UFL2 were screened to better detect the oil-absorbing capacity and flow properties of the powder. Neusilin UFL2 was selected as an adsorbent due to its better oil-absorbing capacity. DSC, X-ray diffraction, and dissolution studies were carried out to characterize the formulation. Further, the Pharmacokinetic profile was also studied in Wistar rats and the Cmax, tmax, and AUC0→t were calculated. The Cmax and AUC0→t plasma concentration is considerably better for the SMEDDS than for the pure drug and marketed formulation. Conclusions: This investigation clearly reveals the potential of developing a solid SMEDDS for candidiasis and invasive aspergillosis treatment, with better efficacy as compared to the commercially available marketed formulation.

## 1. Introduction

In today’s era, nearly 40% of innovative drug candidates show poor water solubility, which poses a challenge during the formulation development of solid oral dosage forms. Several strategies have been utilized recently to solve these issues, such as the usage of micronization, nanoparticles, cyclodextrins, solid dispersion techniques, salt formation, nanoparticles, etc. in drug delivery systems [1]. In recent years, the development of lipid-based drug delivery systems has exponentially improved. Several types of lipid-based formulations exist, such as suspension forms of drugs in lipid, emulsions (*o*/*w* or *w*/*o* emulsion), and more interestingly, self-emulsifying drug delivery systems, self-microemulsifying and self-nano-emulsifying drug delivery systems (SEDDS/SMEDDS/SNEDDS) [2]. When compared to nanoparticles, liposomes, and other drug delivery systems, the voriconazole SMEDDS offers a promising approach due to its ability to improve bioavailability, reduce variability in absorption, and enhance stability without the complexity or potential toxicity seen in other systems. While nanoparticles and liposomes have the advantage of targeted delivery and controlled release, they are more challenging to produce and are associated with higher costs and stability issues. The SMEDDS strikes a balance between efficacy and simplicity, making it a valuable option for antifungal therapy, especially in scenarios where rapid onset, improved bioavailability, and patient compliance are critical [3,4].

Self-emulsifying drug delivery systems (SEDDSs) are an isotropic mixture of oils, surfactant, and cosolvents which emulsify spontaneously to form an oil-in-water emulsion (*o*/*w* emulsion) under gentle stirring when introduced into the aqueous phase system. Recently, self-microemulsifying drug delivery system (SMEDDSs) have attracted increasing interest primarily because they are physically stable formulations which can be manufactured easily [5]. SMEDDSs have an advantage over oily solutions in that they can render a large interfacial area for the partitioning of the drug between the water and oil solution. Solutions, suspensions, and emulsions all are being utilized to increase drug solubility and bioavailability but more recently the primary emphasis has been on the utility of self-microemulsifying drug delivery systems (SMEDDS) within the pharmaceutical industry [6,7].

In recent years, invasive fungal infections have become a very important reason for both morbidity and mortality, especially in immunosuppressed patients. Voriconazole is an antifungal drug, which can be classified under Bio-Classification System (BCS-II) as a drug with low aqueous solubility and high permeability (Biopharmaceutics Classification System). It has low solubility in water, i.e., 0.71 mg/mL. Voriconazole (Vfend), a triazole antifungal drug, is utilized to prevent fungal infections that are usually seen in immunosuppressed patients which comprise invasive candidiasis, *aspergillosis*, and severe fungal infections triggered by *Scedosporium apiospermum* and *Fusarium species*. Voriconazole is the first-line treatment for invasive aspergillosis, a potentially fatal infection caused by Aspergillus species. This infection primarily affects immunocompromised individuals and can lead to high mortality if not treated effectively. Voriconazole has shown superior efficacy compared to amphotericin B, with better survival rates and fewer side effects [8,9].

Several limitations arise, particularly related to the scalability of the formulation process and the relevance of preclinical models (like the Wistar rat model) to human applications. Scaling up the production of voriconazole-loaded SMEDDSs involves challenges in maintaining the formulation stability, achieving batch-to-batch consistency, managing the high surfactant content, and ensuring cost-effective manufacturing. Thorough formulation and production process optimization will be required, in addition to regulatory and safety requirement considerations, to overcome these problems [10].

While the Wistar rat model is a useful tool for preclinical studies, significant differences in GI physiology, metabolism, and disease manifestation between rats and humans limit the direct applicability of preclinical results. To make sure that the pharmacokinetic enhancements and therapeutic efficacy seen in rats translate to positive patient outcomes in humans, more validation in human clinical trials is necessary [11].

Corrective and preventive actions (CAPA) for the use of voriconazole are important to ensure the safe, effective, and optimized treatment of fungal infections, particularly in high-risk patients like those with invasive fungal infections (IFIs) or COVID-19-associated pulmonary aspergillosis (CAPA). CAPA is a key component of the pharmaceutical quality management system, focusing on identifying potential problems, correcting them, and preventing their recurrence in clinical settings. The key to success with voriconazole therapy lies in individualized patient monitoring, the early identification of issues, and proactive management strategies [12]. This study sought to solve the solubility as well as bioavailability issues of voriconazole which belongs to BCS-II by loading it in the form of a liquid SMEDDS and then transforming this into a solid SMEDDS and finally a hard gelatin capsule [5,6].

## 2. Materials and Methods

Voriconazole was obtained from MSN Laboratories Limited, Andhra Pradesh. Labrafil M 1944CS (Gattefosse SAS, Saint-Priest, France), Cremophor RH 40 (BASF Chemicals (Burgbernheim, Germany)), Transcutol HP (Gattefosse SAS, France), and Neusilin UFL2 were obtained from Fuji Chemical Industries, Osaka, Japan.

The purity of voriconazole should be reported by the vendor to ensure the eminence and safety of the product and provided the purity information in the product’s ‘certificate of analysis (CoA)’, which is high purity ≥ 98%.

### 2.1. Solubility Studies

The shake-flask method for determining solubility was used to conduct the voriconazole solubility investigations. Two grams of oil, the oil–surfactant mixture, and the oil–co-surfactant mixture were each given an excess of voriconazole in 5 mL capacity stop-per vials. Following a 10-min vortex shaker, the vials were then put in an orbital shaker set to 25 ± 1 °C for 72 h. The equilibrated samples were re-moved and centrifuged at 4000 rpm for 20 min. A 0.45 µm membrane filter was used to filter the resultant supernatant layer. Using a UV spectrophotometer, the concentration of voriconazole was measured at a wavelength of 256 nm [13].

### 2.2. Preparation of Pseudo-Ternary Phase Diagram

Oils, surfactants, and co-surfactants were chosen for voriconazole based on solubility experiments, and ternary phase diagrams of different combinations of these combinations were subsequently created. Using the water titration approach, the pseudo-ternary phase diagram for the oil, water, and surfactant mixture (Smix) was created. The Smix ratio was further adjusted at various weight-based ratios. Nine distinct transparent and uniform mixtures of oil, either surfactant or oil, were used for each phase diagram. Smix at ratios of 1:9, 2:8, 3:7, 4:6, 5:5, 6:4, 7:3, 8:2, and 9:1 were created by gently mixing using a magnetic stirrer. Next, a micropipette was used to carefully titrate the oil–surfactant mixture (Smix) with water [14]. The mixtures were vortexed for two to three minutes after each addition, and then they were let to remain at room temperature for fifteen minutes. After equilibration, the mixtures were visually assessed for transparency. The point at which the mixture became opaque or showed signs of phase separation was measured as the end point of the titration. The trapezoidal method was used for calculating the area under the microemulsion region.

### 2.3. Preparation of SMEDDS

This technique is especially useful for drugs like voriconazole, which have low solubility, and can improve the therapeutic efficacy by enhancing bioavailability and ensuring consistent drug delivery. The use of solid carriers can further enhance the stability and convenience of the formulation for oral administration. In our study, Labrafil M 1944 CS was selected as the oil, Cremophor RH 40 as the surfactant, and Transcutol HP as the co-surfactant, respectively. In short, oil was added to previously weighed voriconazole (unit dose 50 mg). Thereafter, components were kept in a sonicator (for 20–25 min., frequency 20–40 kHz) at a certain temperature (40–50 °C) until the drug was completely solubilized in the lipid phase. Then, the surfactant and co-surfactant were added to the prepared composition and emulsified using a magnetic stirrer (30–40 min.) until a clear emulsion was formed [15,16].

### 2.4. Characterization and Evaluation of Liquid SMEDDS

#### 2.4.1. Self-Emulsification and Dispersibility Studies

Using a USP type-II dissolution device, the self-emulsification efficiency of liquid SMEDDS was determined. Subsequently, 500 mL of distilled water was mixed with 1 mL of the optimized liquid SMEDDS formulation at 37 ± 0.5 °C and 50 rpm with gentle stirring [10]. The SMEDDS formulation’s in vitro concert was assessed visually using the grading method that is displayed in Table 1.

#### 2.4.2. Robustness of Dilution

In this method, optimized liquid SMEDDS were subjected to various diluents in different ratios i.e., 1:50, 1:100, and 1:250. Various diluents like H_2_O, 0.1 N HCL, and phosphate buffer (pH 6.8) were utilized for the assessment of the robustness of the dilution. The diluted emulsions were observed after 1 h, 2 h, 6 h, 24 h, and 48 h for the detection of the phase separation or precipitation of the drug [17]. The formulations which did not reveal any sign of phase separation were considered for further study.

#### 2.4.3. Droplet Size Determination

After preparation of liquid SMEDDS, the estimation of the droplet size is necessary. The optimized liquid formulation was prefiltered with a millipore 0.45 μm filter. Then, 0.1 g of prepared SMEDDS was diluted with 10 mL of double-distilled water. The droplet size was determined after being diluted 100 times using Malvern Zetasizer Nano (Malvern Instruments, Malvern, UK) [18].

#### 2.4.4. Zeta Potential Determination

The zeta potential of the liquid SMEDDS was evaluated using a suitable Malvern Zetasizer Nano (Malvern Instruments, UK). Zeta potential is mainly used for the identification of the charge present on droplets. In all the liquid SMEDDS formulations, the zeta potential had a negative value [19].

#### 2.4.5. Viscosity Determination

To find the liquid SMEDDS’s viscosity, a Brookfield DVE viscometer (Brookfield Engineering Laboratories, Inc., Middleboro, MA, USA) was used. A liquid portion of approximately 0.5 g was obtained for analysis at 100 rpm at 25 ± 1 °C without any dilution [4]. The viscosity was found.

#### 2.4.6. Percentage Transmittance

Dilution of the optimized formulation (1 mL) in 100 mL of distilled water was carried out with continuous stirring using a magnetic stirrer running at 50 rpm. Using distilled water as a blank, the percentage transmittance was measured at 560 nm using a UV-visible spectrophotometer [20].

#### 2.4.7. Thermodynamic Stability Studies

##### Heating Cooling Cycle

The liquid SMEDDS underwent a cycle of heating and cooling. For two days, a refrigerator with a temperature range of 4 °C to 45 °C was used to complete this cycle.

##### Centrifugation

The microemulsion generated on a dilution of liquid SMEDDS was centrifuged at 3500 rpm for 30 min using Centrifuge 5810 R, Hamburg. The freeze–thaw test was permitted for those formulations that showed no evidence of phase separation.

##### Freeze–Thaw Cycle

The liquid SMEDDSs underwent a 48-h analysis period during which they were frozen at −4 °C and thawed at 40 °C. Following the completion of the freeze–thaw cycle, visual inspection was necessary [21].

### 2.5. Characterization and Evaluation of a Solid SMEDDS

#### 2.5.1. Drug Content Estimation

Using the UV technique, the voriconazole content in a solid SMEDDS was ascertained. Enough methanol was used to dissolve the liquid SMEDDS. After 10 min of sonication, the solution was filtered out. The filtrate’s absorbance was measured using a UV-visible spectrophotometer at 256 nm.

#### 2.5.2. Flow Properties

The prepared solid SMEDDS formulations were evaluated for various flow properties such as bulk density, tapped density, Hausner’s ratio, Carr’s index, and angle of repose.

#### 2.5.3. Differential Scanning Calorimetry (DSC)

The physical state of the solid SMEDDS was detected by using differential scanning calorimetry (DSC, Mettler Toledo, Greifensee, Switzerland). Samples of about 4–5 mg were taken and hermetically sealed in aluminum pans under the atmosphere of nitrogen [N_2_] at a constant heating rate of 10 °C/min. The samples were heated over a temperature range of 40 °C to 200 °C [22].

#### 2.5.4. X-Ray Powder Diffraction (XRD)

The voriconazole solid SMEDDS’s physical condition was assessed using an X-ray diffractometer (PAN analytical, Budel, The Netherlands). Cu served as an anode tube, and a high voltage of roughly 45 kv and a current of 40 mA were applied. The samples were further subjected to a Cu-K radiation at a regular speed of 2θ/min. Following a 15-min dwell period, X-ray data were gathered at a pace of one second for each step. This demonstrated if the powder sample was crystalline or amorphous [23].

#### 2.5.5. ATR-FTIR Spectroscopy

The ATR-FTIR spectra of the pure drug, adsorbent, and the optimized formulation were examined using an ATR-FTIR spectrophotometer (ATR-FTIR Perkin Elmer FTIR-100). The ATR-FTIR spectra were obtained by employing the potassium bromide pressed disc method. The scanning range was 400-4000 cm^−1^.

#### 2.5.6. Morphology Study by SEM

The solid voriconazole SMEDDS, a pure medication, was examined morphologically using a scanning electron microscope (JSM-6510, Jeol, Tokyo, Japan).

### 2.6. In Vitro Release Study

Size 00 firm gelatin capsules containing the optimized solid SMEDDS formulation were packed. Additional in vitro dissolution investigations were conducted on the firm gelatin capsules. The USP type-II apparatus was utilized to conduct the dissolution test. The dissolution medium in this case was 0.1 N HCl, and a 50-rpm speed and 37 ± 0.5 °C temperature were maintained. Samples of 5 mL were taken out at intervals of 5, 10, 20, 30, 40, and 60 minutes. A UV-visible spectrophotometer set to 256 nm was used to further analyze the samples that had been collected [24].

### 2.7. Stability Studies

The solid SMEDDS formulation was filled in empty hard gelatin capsules flushed with nitrogen gas and finally sealed in HDPE bottles. The samples were subjected to stability studies at different temperature conditions, i.e., 25 °C and 60% relative humidity, 30 °C and 65% relative humidity, as well as 40 °C and 75% relative humidity [25].

### 2.8. Pharmacokinetic Study

Wistar rats of either sex (180–250 g) were employed in this study. They were brought from Panacea Biotec Ltd., Lalru, Punjab, India and had free access to water and food. The experimental protocol was approved by the Institutional Animal Ethics Committee (Approval no. 107/GO/ReBi/S/99/CPCSEA/2018-11).

Wister rats were utilized as an animal model for conducting animal pharmacokinetic studies. The Wistar rats, with a weight of approximately 250.0 ± 10 g, were separated into three different groups i.e., the control formulation, test formulation, and marketed formulation. The dose treatment was done by the oral route using oral gavage tubes. Samples of blood were immediately harvested before the drug administration at (time zero) and after the drug administration at different time intervals, i.e., 0.5, 0.75, 1, 1.5, 2, 4, 6, 8, 10, and 12 h of dosage treatment. Thereafter, the anticoagulant agent, i.e., heparin, was mixed with the blood samples to prevent the coagulation of blood. The harvested blood samples were further kept in eppendorf tubes for centrifugation at 4000 r.p.m. for 15 to 20 min. After centrifugation, rat plasma was obtained and the concentration of voriconazole in rat plasma was estimated using the HPLC method [26].

## 3. Results

### 3.1. Solubility Studies

Solubility studies were carried out to detect the suitable oil phase, surfactant, and co-surfactant for the development of the voriconazole SMEDDS. The solubility of voriconazole in various oils, surfactants, and co-surfactants is given in Table 2. Herein, Labrafil M 1944CS was selected as the oil, Cremophor RH 40 and Transcutol HP were selected as the surfactant and co-surfactant based on solubility studies and due to the high solubility of drug in these excipients. The maximum solubility of voriconazole was found to be 126.38 ± 3.18 mg/mL in Labrafil M 1944CS (oil), 128.86 ± 2.94 mg/mL in Cremophor RH 40 (surfactant), and 139.19 ± mg/mL in Transcutol HP (co-surfactant). The minimum solubility of voriconazole was 7.48 ± 3.65 mg/mL in olive oil, 33.58 ± 2.72 mg/mL in span 20 (surfactant), and 50.46 ± 4.42 mg/mL in plurololeique (co-surfactant). On the basis of solubility studies, Labrafil M 1944CS, Cremophor RH 40, and Transcutol HP were selected for further studies.

### 3.2. Pseudo-Ternary Phase Diagram

Pseudo-ternary phase diagrams are essential tools used to optimize self-microemulsifying drug delivery system (SMEDDS) formulations by identifying the optimal ratios of oil, surfactant, and co-surfactant to form stable microemulsions. The primary goal of constructing a pseudo-ternary phase diagram is to identify the microemulsion region where a stable and clear microemulsion is formed. Another goal is to determine the optimal ratios of oil, surfactant, and co-surfactant that result in a thermodynamically stable microemulsion with the smallest possible droplet size. Optimizing the self-emulsifying properties of the formulation ensures quick and uniform emulsification when the formulation comes into contact with aqueous fluids (e.g., gastrointestinal fluids).

**Figure 1:**
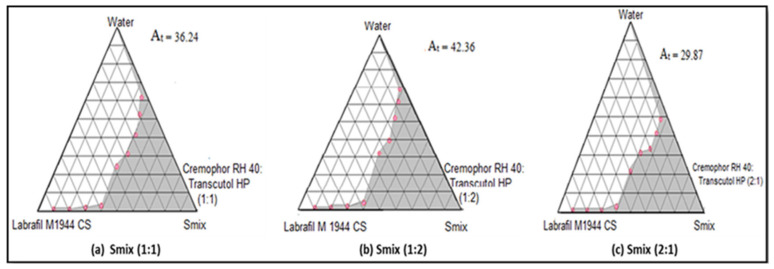
Pseudo-ternary phase diagrams containing oil (Labrafil M 1944 CS), S_mix_ (surfactant: Cremophor RH 40; co-surfactant: Transcutol HP), and water at different S_mix_ ratios i.e., (**a**) 1:1 (**b**) 1:2 (**c**) 2:1.

To construct a pseudo-ternary phase diagram, Labrafil M 1944 CS (oil), Cremophor RH 40 (surfactant), and Transcutol HP (co-surfactant) were selected as the components of the SMEDDS based upon the solubility studies. The self-microemulsion area was found to be greater for formulation F2S (1:2), as shown in Figure 1. It has been reported that a single surfactant might not be able to reduce o/w interface compulsorily. Therefore, it is assumed that the combination of short to medium chain-length alcohols (e.g., Transcutol HP) with single-chain surfactants could further result in lowering the interfacial tension. However, with the use of medium chain-length alcohols, aqueous as well as oil phase miscibility could be improved due to the partitioning behavior between the two phases [16].

In this study, microemulsions (MEs) were spontaneously formed at 25° ± 0.5° C when their components were brought into contact. Phase diagrams of MEs were constructed to detect the ME existence zone. Here, the surfactant/co-surfactant mixtures with altered weight ratios were developed by mixing Cremophor.

RH 40 and Transcutol HP. Cremophor RH 40 and Transcutol HP in weight ratios of 1:2 showed a maximum ME area of 42.36 (Figure 1). The ME area of different Smix ratios is shown in Table 3. The maximum ME area was observed with a 1:2 surfactant—co-surfactant mixture and hence this composition of Smix was used for further studies.

### 3.3. Self-Emulsification and Dispersibility Studies

SMEDDSs are thermodynamically stable formulations and are formed at a fixed concentration of oil, surfactant, and water, with no phase separation, creaming, or cracking. It is the thermostability which differentiates between nano or microemulsion from emulsions that have kinetic stability and will ultimately phase separate [17]. Thereafter, the selected formulations were subjected to different thermodynamic stability tests, such as heating cooling cycle, centrifugation, and freeze–thaw cycle stress tests.

Those formulations that passed the thermodynamic stability tests were taken for a dispersibility test. It was found that formulations F6S and F7S did not pass the thermodynamic stability tests and thus were dropped for further study. Herein, formulations are considered to be Grade A and Grade B on the basis of their appearance and clarity, as mentioned in Table 3. Formulations that cleared the dispersibility test in Grades A and B were taken for further study, as Grade A and B formulations will stay as microemulsions when dispersed in GIT, whereas other formulations falling in the category of Grades C and D were discarded for further study. The results of the self-emulsification and dispersibility studies are depicted in Table 3.

### 3.4. Viscosity Determination

The viscosities of the various liquid part formulations were assessed by using a Brookfield DVE viscometer (spindle no. S-34) at 100 rpm. It has been observed that the viscosity of the MEs increased along with increase in the amount of Smix. The optimized formulation F2S exhibited minimum viscosity of 33 ± 2.23 cps. It has been reported that the viscosity varied from 11.06 ± 0.48 cps to 18.04 ± 0.53 cps in VCZ-ME whereas in the voriconazole SEDDS the viscosity of optimized formulation was found to be 32 ± 2.79 cps.

### 3.5. Percentage Transmittance

The clarity of the microemulsions was examined by transparency and measured in terms of transmittance (%T). SMEDDS forms *o*/*w* microemulsion since water is external phase. All the formulations were assessed out for the % transmittance and the value lies between 91.27–98.28%. Formulation F2S has % transmittance value greater than 98% which indicates high clarity of microemulsion. The % transmittance of the optimized formulation was found to be 98.28% which indicates that the formulation was more transparent in comparison to that of various other formulations.

### 3.6. Characterization of Solid SMEDDSs

#### 3.6.1. Drug Content Estimation

The drug content of all the solid SMEDDS formulations was found to be between 88.78 and 99.56%. The optimized formulation F2S (1:2) showed a maximum amount of drug content of 99.56%. The results are shown in Table 4.

#### 3.6.2. Flow Properties

Tests of the flow properties of various solid SMEDDS formulations were performed and the results are shown in Table 4. The results of Hausner’s ratio, Carr’s index, and angle of repose depicted that all the formulations have good flow properties, with F2S exhibiting the best. The formulation with the maximum drug content has a 28.38 ± 2.48 angle of repose, with excellent flow properties. On the basis of these studies, the F2S formulation has increased flow properties and was selected as the optimized formulation, and all the further investigations were carried out on F2S only.

#### 3.6.3. Droplet Size Determination

The particle size of the optimized batch was observed to be 19.04 nm and the polydispersity index (PDI) value was found to be 0.162. It has been reported that the smaller the particle size of the emulsion droplet, the more rapid the absorption and the bioavailability of the formulation [27]. An investigation was carried out by Bhosale-et-al on voriconazole using oleic acid, isopropyl myristate, and isopropyl palmitate, and the droplet size was found to be 160.1 ± 3.9 nm to 245.2 ± 2.90 nm for oleic acid, 158.1 ± 2.13 nm to 203.2 ± 2.78 nm for isopropyl myristate, and 152.3 ± 3.06 nm to 241.4 ± 3.24 nm for isopropyl palmitate, where all the formulations show a droplet size of below 250 nm.

#### 3.6.4. Zeta Potential Determination

The zeta potential actually controls the microemulsion stability; it is necessary to determine its value for stability samples. The charge of SMEDDSs is a crucial factor which should be assessed properly. The optimized formulation’s zeta potential value was observed to be −22.7 mV. It was also investigated by a researcher that zeta potential decreases with the increase in surfactant concentration and the zeta potential of the selected formulation was found to be −13.6 in the voriconazole SEDDS. It has been assessed that a dividing line between stable and unstable aqueous dispersions is usually taken at either +30 or −30 mV, which suggests that particles with a zeta potential of higher than +30 mV or within −30 mV could be considered as stable [28].

#### 3.6.5. Differential Scanning Calorimetry (DSC)

**Figure 2:**
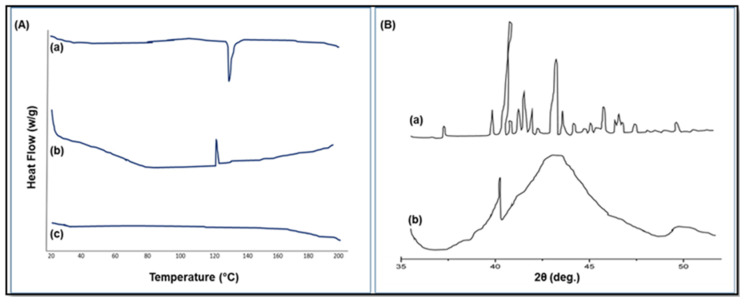
(**A**): DSC thermogram of pure drug (a), placebo physical mixture (b), and S-SMEDDS (c); (**B**): X-ray diffractogram of voriconazole (a), X-ray diffractogram of solid SMEDDS (b).

The pure drug showed a sharp endothermic peak at 131.56 °C corresponding to its melting point and the heat of fusion was found to be 96.60 J/g [Figure 2A(a)]. Such a sharp endothermic peak implies that a plain drug used was in an absolutely pure crystalline state. However, in the DSC thermogram of the placebo physical mixture of the solid SMEDDS, an endothermic peak at 120.82 °C was observed, which corresponds to the melting point of Neusilin UFL2 used as an adsorbent in the solid SMEDDS [Figure 2A(b)]. On the other hand, no obvious peaks were observed in the case of the solid SMEDDS of voriconazole, which indicates that the drug was completely dissolved, molecularly dispersed within the S-SMEDDS matrix and must be present in an amorphous state [Figure 2A(c)]. This disappearance of the drug peaks upon the formulation of the SMEDDS may be due to the formation of an amorphous solid state [29,30].

#### 3.6.6. X-Ray Powder Diffraction (XRD)

An X-ray diffraction study was carried out to ascertain the physical state of voriconazole pure drug and that of the optimized formulation. The X-ray diffraction of pure drug showed sharp diffraction peaks which predict the crystalline nature of the drug. However, the X-ray diffraction of the optimized formulation illustrates the amorphous state of the drug in the formulation (F2S), as shown in Figure 2B.

#### 3.6.7. ATR-FTIR Spectroscopy

**Figure 3:**
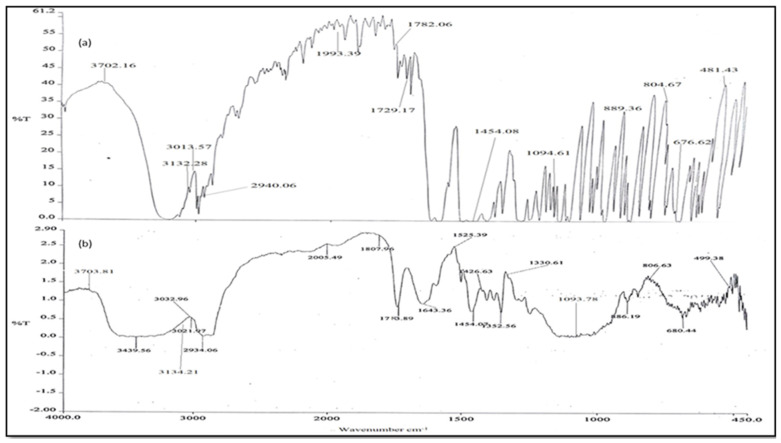
ATR-FTIR spectra: (**a**) voriconazole (**b**) optimized formulation.

The ATR-FTIR spectra of the pure drug and the optimized formulation (F2S) were examined using an ATR-FTIR spectrophotometer. The characteristic absorption peaks obtained were of C-O at 1782.06 cm^−1^, O-H at 3702.16 cm^−1^, N-H at 3132.28 cm^−1^, and S=O at 1094.61cm^−1^. The peaks observed were similar to those reported in the literature. However, in the optimized formulation similar peaks were observed, whereas the interfering peaks were absent, which indicates that there was no interaction between the drug and other excipients utilized during the study, as shown in Figure 3.

#### 3.6.8. SEM Analysis

**Figure 4:**
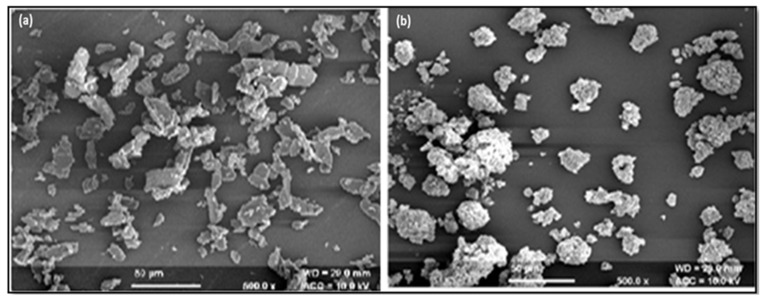
SEM images of (**a**) pure VCZ, (**b**) SMEDDS at 500×.

The SEM analysis showed that S-SMEDDS appeared to be smooth surfaced, indicating that the liquid SMEDDS is adsorbed or embedded into the pores of Neusilin UFL2 with a smaller amount of aggregation. Herein, the SEM analysis at 500× showed the crystalline state of the pure drug (Figure 4a), whereas an amorphous state appeared in the S-SMEDDS formulation (Figure 4b).

### 3.7. In Vitro Release Study

**Figure 5:**
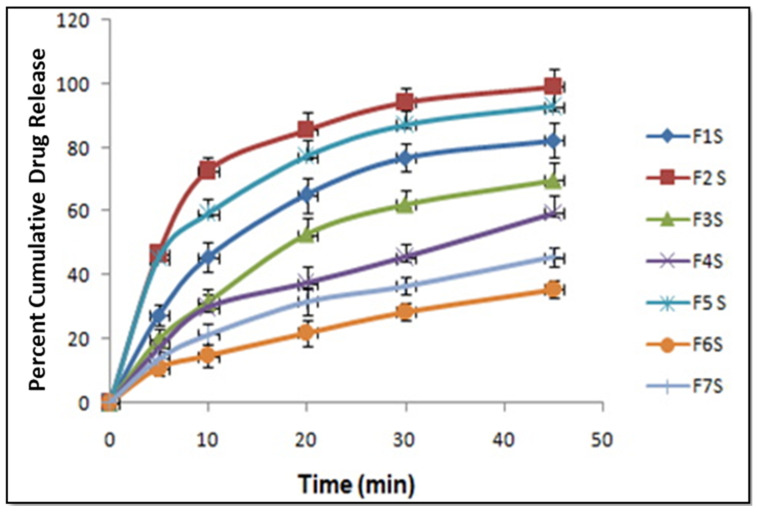
Percent cumulative drug release of solid SMEDDS formulation (data expressed as mean ± S.D, *n = 3*).

In vitro dissolution studies were carried out using USP type-II dissolution apparatus (Paddle type) for all the formulations. In vitro dissolution studies were conducted in 0.1 N HCL dissolution media. It was observed that formulation F2S (1:2) exhibited a high percentage of cumulative drug release in comparison to that of other formulations. The in vitro drug release of various solid SMEDDS formulations is shown in Figure 5. Oneway ANOVA was applied for comparison of the in vitro drug release, which showed that there is a significant difference between the different formulations i.e., *p* < 0.05.

### 3.8. Stability Studies

On the basis of the above study and the characterization of the solid SMEDDS, it was found that formulation F2S (1:2) was the optimized formulation, and a stability study was conducted on it. The stability study was performed as per the conditions set out in the ICH guidelines, based on that particular zone. A real-time stability study (25 ± 2 °C/60 ± 5% RH), long-term stability study (30 ± 2 °C/65 ± 5% RH), and accelerated stability study (40 ± 2 °C/75 ± 5% RH) were conducted on the formulation F2S (1:2) for a period of one and three months. The results of the real-time stability studies (25 ± 2 °C/60 ± 5% RH), long-term stability studies (30 ± 2 °C/65 ± 5% RH), and accelerated stability studies (40 ± 2 °C/75 ± 5% RH) are shown in Table 5.

### 3.9. Pharmacokinetic Studies

Comparing voriconazole-loaded SMEDDS to traditional formulations, the Cmax is often higher. This is because the medication is more soluble and absorbs more quickly in the gastrointestinal (GI) tract, resulting in a tmax take a quicker time to peak concentration for the medication. For circumstances in which rapid antifungal action is required, the speedy development of a microemulsion in the GI fluids facilitates faster drug release and absorption. A greater AUC denotes better total drug exposure, which translates to more medication being absorbed gradually into the circulation.

**Figure 6:**
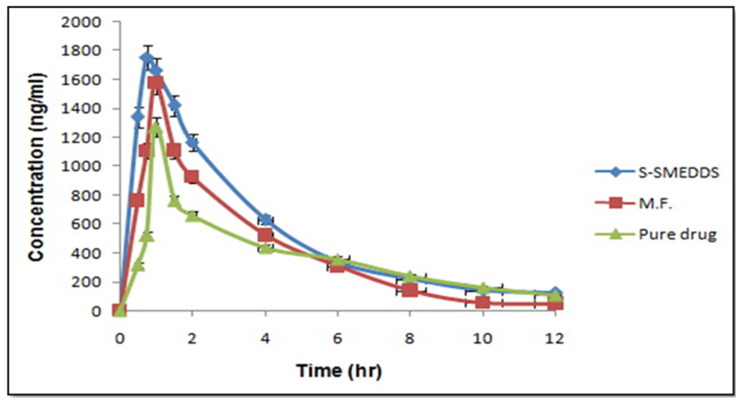
In vivo comparison of S-SMEDDS marketed formulation and pure drug. The results were analyzed by two-way ANOVA followed by a bonferroni posthoc test; all the samples were compared with the rest of the samples (when compared with M.F.; when compared to pure drug); (Data expressed as mean ± S.D, *n* = 3).

Voriconazole’s in vivo pharmacokinetic study using S-SMEDDS (F2S), commercial formulation, and pure medication were investigated in rats. The average plasma concentration was graphed against time, as illustrated in Figure 6. The pharmacokinetic parameters of voriconazole absorption were assessed using the non-compartment model. The area under curve (AUC) was computed using the linear trapezoidal rule. Compared to the pure drug and the commercial formulation, S-SMEDDS’s plasma concentration Cmax and AUC0→t were significantly superior. Tmax was lowered for S-SMEDDS and was 0.75 h for the marketed formulation and 1 h for the pure medication. These results predicted the feasibility of SMEDDS as a better delivery system. The pharmacokinetic profile of voriconazole-loaded SMEDDS indicates substantial improvements in drug absorption, bioavailability, and therapeutic potential compared to conventional formulations. SMEDDS enhances the solubility and dissolution of voriconazole, leading to higher and faster peak plasma concentrations, increased overall drug exposure, and better antifungal efficacy. These improvements are particularly important for treating systemic fungal infections, where rapid and effective drug delivery is critical.

## 4. Discussion

Solid microemulsion preconcentrates containing voriconazole offer several promising clinical benefits, including enhanced bioavailability, reduced toxicity, more predictable drug levels, and potentially improved patient outcomes in the treatment of severe fungal infections. These benefits make this formulation an exciting advancement in antifungal therapy, particularly for immunocompromised patients or those with conditions like COVID-19-associated pulmonary aspergillosis (CAPA), where prompt and effective treatment is critical. However, challenges related to cost, manufacturing, and the need for further clinical validation should be considered.

The advantage of voriconazole-loaded SMEDDS lies in its ability to address several challenges associated with the conventional administration of voriconazole and other antifungal drugs, particularly in terms of bioavailability, solubility, and pharmacokinetic variability.

As an antifungal medication, voriconazole falls under BCS-II medication classification. In this study, an attempt has been made to resolve voriconazole’s solubility and bioavailability problems by putting the drug into liquid SMEDDS, which subsequently solidifies into a hard gelatin capsule. In order to determine the ideal oil phase, surfactant, and co-surfactant for the development of the voriconazole SMEDDS, voriconazole solubility tests were conducted. Voriconazole is soluble in several types of oils, co-surfactants, and surfactants. Labrafil M 1944CS, Cremophor RH 40, and Transcutol HP were chosen for additional research based on solubility experiments [31]. Other antifungal agents like itraconazole and posaconazole also suffer from low solubility. While they have been formulated into different delivery systems (e.g., nanoparticle suspensions, cyclodextrin-based solutions), the SMEDDS provides an advantageous alternative by eliminating the need for external solubilizers or surfactants, improving both ease of use and patient compliance.

Pseudo-ternary phase diagrams have been created for optimization. Labrafil M 1944 CS (oil), Cremophor RH 40 (surfactant), and Transcutol HP (co-surfactant) were used in order to create the pseudo-ternary phase diagram. For formulation F2S (1:2), it was discovered that the self-microemulsion region was larger. There have been tidings that o/w interface reduction may not be the exclusive domain of a single surfactant. Thus, it stands to reason that the interfacial tension may be further reduced by combining short- to medium-chain alcohols with single-chain surfactants. However, because of the way the two phases partition, miscibility in both the oil and aqueous phases may be enhanced by the use of medium-chain-length alcohols [32].

The prepared SMEDDS are thermodynamically stable formulations that are created at a set concentration of oil, surfactant, and water, with no phase separation, creaming, or cracking. Following that, many thermodynamic stability tests, including centrifugation, freeze–thaw cycle stress testing, and heating–cooling cycle tests, were performed on the selected formulations. Furthermore, the viscosity and % transmission of the microemulsion were determined. After being transformed from a microemulsion into a solid SMEDDS, the drug content, flow characteristics, droplet size, zeta potential, DSC, X-ray, FTIR, SEM, in vitro release, stability, and pharmacokinetic analysis were assessed [33].

The selected formulation had improved flow characteristics such as Hausner’s ratio, Carr’s index, and angle of repose, with a high drug content. The optimized batch’s particle size was found to be nano size, in line with the theory that faster absorption and higher bioavailability of the formulation occur when emulsion droplets have smaller particle sizes. The medicine and excipients do not interact in any way, according to the DSC, X-ray, and FTIR studies. The SMEDDS appeared to have a smooth surface, according to the SEM study, suggesting that the liquid SMEDDS is embedded or adsorbed into the Neusilin UFL2 pores with a lesser degree of aggregation. The USP type-II dissolution device was used for in vitro release experiments. When compared to other formulations, formulation F2S was shown to have a high percentage of cumulative drug release. Based on the conditions specified by the ICH for that specific zone, stability research was carried out. Very little size, zeta potential, and drug content are impacted in all three zones—real time, long term, and accelerated stability [34].

Other antifungals like fluconazole have higher bioavailability but are limited in their antifungal spectrum. Voriconazole’s SMEDDS provides both enhanced bioavailability and a broad spectrum of antifungal activity, offering a potent alternative to fluconazole for more serious fungal infections like invasive *aspergillosis.* Antifungals like amphotericin B are effective but have significant toxicity concerns. SMEDDS, being an oral and less toxic alternative, enhances patient adherence compared to intravenous amphotericin B, making it more suitable for long-term treatment.

Rats were used in an in vivo pharmacokinetic investigation of voriconazole with S-SMEDDS (F2S), the commercial formulation, and the pure medication. The pharmacokinetic parameters of voriconazole absorption were assessed using the non-compartment model, and the results indicate that SMEDDSs may be a more viable administration method. When compared to commercially available marketed formulations, SMEDDSs exhibit superior efficacy.

## 5. Conclusions

Fungal infections still exist as a critical reason of morbidity and mortality despite significant advances in science and medicine. Recently, azoles have been the most broadly used class of antifungal drugs. A voriconazole SMEDDS was successfully prepared and evaluated. From the preliminary studies, it was revealed that the amount of oil, surfactant, and co-surfactant were critical parameters in determining the particle size of the formulation. Based upon solubility studies, Labrafil M 1944 CS was chosen as the oil, Cremophore RH 40 as the surfactant, and Transcutol HP as the co-surfactant. The moving of a SMEDDS preconcentrate into the aqueous media under gentle stirring results in the formation of a translucent oil in-water microemulsion. The particle size of the optimized formulation F2S (1:2) was observed to be 19.04 nm and the polydispersity index (PDI) value was found to be 0.162. The optimized formulation zeta potential value was observed to be −22.7 mV. The particles did not show signs of any flocculation and the formulation was observed to be steady.

Selected formulations were subjected to stress conditions i.e., freeze–thaw, heating and cooling cycle tests. Further, the optimized liquid SMEDDS was converted into a solid SMEDDS. Then, the powder flow properties of the solid SMEDDS was assessed. After that, the solid SMEDDSs were filled into hard gelatin capsules. The assay content indicates uniform dispersion of the drug in solid SMEDDSs. A pharmacokinetic study was assessed further after oral administration of the drug to rats. An in vivo standard plot was developed and it was observed that the S-SMEDDS showed better drug release as compared to the pure drug and marketed dosage forms. The encouraging results obtained from the above investigation reveal strong evidence of the promising role of developing solid SMEDDS for candidiasis and invasive aspergillosis treatment, with better efficacy as compared to commercially available marketed formulations. However, it is necessary to carry out scale-up studies to detect the feasibility of producing solid SMEDDSs on an industrial scale.

Voriconazole-loaded SMEDDSs present a promising approach to improving the clinical management of systemic fungal infections by enhancing the bioavailability, consistency, and tolerability of voriconazole. Human models, through pharmacokinetic studies, clinical trials in patients with fungal infections, and studies in high-risk and special populations, will play a critical role in demonstrating the clinical utility of this formulation.

## Figures and Tables

**Table 1:** Grading system for assessment of dispersibility of microemulsion.

Assessment of Dispersibility	Self-Emulsification Time	Grade
Rapidly forming, clear or slightly bluish form	Less than 1 min	A
Rapidly forming, bluish or slightly turbid in appearance	Less than 2 min	B
Rapidly forming, milky emulsion in appearance	Less than 2 min	C
Slowly forming, with grayish white emulsion along with oily appearance on upper surface	More than 2 min	D
Inadequate or insignificant emulsification combined with surface-level oil globules	More than 3 min	E

**Table 2:** Solubility analysis of voriconazole in various oils, surfactants, and co-surfactants.

S. No	Oils	Solubility (mg/mL)	Cremophore Rh40: Transcutol	ME Area	Surfactants	Solubility
1.	Oleic acid	108.63 ± 2.84	1:1	36.24	Cremophore RH 40	128.86 ± 2.94
2.	Isopropyl Myristate	80.01 ± 5.60	1:2	42.36	Cremophore EL	77.33 ± 6.68
3.	Capmul MCM	86.07 ± 3.28	2:1	29.87	Tween 20	121.64 ± 2.07
4.	Olive oil	7.48 ± 3.65	1:4	20.23	Tween 80	66.45 ± 3.48
5.	Labrafil M 1944CS	126.38 ± 3.18	4:1	13.56	Span 20	33.58 ± 2.72
6.	Arachis oil	59.29 ± 2.82	1:3	33.19		
7.	Maisine 35-1	92.91 ± 3.90	3:1	21.37		

**Table 3:** Various evaluation parameters of different formulations of SMEDDSs.

Formulation Code	Self-Emulsification Assessment	Viscosity (cps)	Assessment of Dispersibility	Robustness of Dilution	Centrifugation Test (Phase Separation)	Freeze–Thaw Method
F1S	Grade A	32 ± 2.62	Clear, rapidly forming	Pass	No	No change
F2S	Grade A	33 ± 2.23	Clear, rapidly forming	Pass	No	No change
F3S	Grade A	37 ± 4.98	Clear, rapidly forming	Pass	No	No change
F4S	Grade B	42 ± 4.69	Bluish, slightly turbid	Pass	No	No change
F5S	Grade A	35 ± 3.12	Clear, rapidly forming	Pass	No	No change
F6S	Grade C	43 ± 2.83	Rapidly forming, milky appearance	Fail	Yes	Change
F7S	Grade D	47 ± 3.38	Slowly forming, grayish white emulsion	Fail	Yes	Change

**Table 4:** Evaluation parameters of solid SMEDDSs.

Formulation Code	Drug Content	Angle of Repose	Bulk Density gcm^−3^	Tapped Density gcm^−3^	Hausner’s Ratio gcm^−3^	Carr’s Index %
F1S	92.34 ± 2.71	28.92 ± 2.95	0.34	0.43	1.26	20.93
F2S	99.56 ± 0.17	28.38 ± 2.48	0.30	0.36	1.20	16.66
F3S	89.39 ± 2.12	31.29 ± 1.32	0.34	0.45	1.32	24.44
F4S	93.21 ± 1.92	34.58 ± 1.56	0.32	0.44	1.37	27.27
F5S	94.89 ± 1.64	30.18 ± 1.83	0.33	0.42	1.27	21.42
F6S	90.23 ± 2.58	35.21 ± 2.31	0.36	0.45	1.25	20.45
F7S	88.78 ± 2.83	41.98 ± 3.98	0.37	0.51	1.37	27.45

**Table 5:** Results of real-time stability studies at 25 ± 2 °C/60 ± 5% RH.

Real-Time Stability Studies at 25 ± 2 °C/60 ± 5% RH				
Sampling Time (Months)	Droplet Size (nm)	Zeta Potential (mV)	Drug Content	Viscosity (cps)
0	19.04 ± 1.12	−22.7 ± 0.3	99.56 ± 0.17	33 ± 2.23
1	20.89 ± 1.26	−21.8 ± 0.5	99.23 ± 0.12	34 ± 1.39
3	22.63 ± 1.92	−20.7 ± 0.7	98.87 ± 0.09	35 ± 1.43
**Long-term stability studies at 30 ± 2 °C/65 ± 5% RH**				
0	19.04 ± 1.12	−22.7 ± 0.3	99.56 ± 0.17	33 ± 2.23
1	22.67 ± 1.77	−20.1 ± 0.4	98.21 ± 0.14	34 ± 2.98
3	23.78 ± 1.91	−19.4 ± 0.6	97.73 ± 0.13	37 ± 3.76
**Accelerated stability studies at 40 ± 2 °C/75 ± 5% RH**				
0	19.04 ± 1.12	−22.7 ± 0.3	99.56 ± 0.17	33 ± 2.23
1	25.15 ± 1.29	−18.4 ± 0.8	97.56 ± 0.13	35 ± 2.96
3	26.72 ± 2.19	−17.2 ± 0.7	96.62 ± 0.11	39 ± 3.17

## Data Availability

The data will be available from the authors upon reasonable request.
